# Trends in Risk Factors for Cardiovascular Disease Among Iranian Adolescents: The Tehran Lipid and Glucose Study, 1999–2008

**DOI:** 10.2188/jea.JE20100162

**Published:** 2011-09-05

**Authors:** Firoozeh Hosseini-Esfahani, Ateke Mousavi Nasl Khameneh, Parvin Mirmiran, Arash Ghanbarian, Fereidoun Azizi

**Affiliations:** 1 Obesity Research Center, Research Institute for Endocrine Sciences, Shahid Beheshti University of Medical Sciences, Tehran, Iran; 2 Department of Human Nutrition, Faculty of Nutrition and Food Technology, National Nutrition and Food Technology Research Institute, Shahid Beheshti University of Medical Sciences, Tehran, Iran; 3 Endocrine Research Center, Research Institute for Endocrine Sciences, Shahid Beheshti University of Medical Sciences, Tehran, Iran

**Keywords:** adolescents, cardiovascular risk factors, hyperlipidemias, hypertension, overweight

## Abstract

**Objectives:**

Data on secular trends in adolescent obesity and dyslipidemia are limited. Data on obesity status collected during 3 surveys were used to evaluate these trends in obesity and dyslipidemia among Tehranian adolescents and to assess the likelihood of risk factors for cardiovascular disease.

**Methods:**

We analyzed data for adolescents (age 10 to 19 years) from 3 cross-sectional surveys of the Tehran Lipid and Glucose Study: 1999–2001 (*n* = 3010, 47.2% males), 2002–2005 (*n* = 1107, 48.4% males), and 2006–2008 (*n* = 1090, 46.6% males). Overweight and abdominal obesity were defined using Iranian body mass index (BMI) percentiles, International Obesity Task Force (IOTF) criteria, and Iranian waist circumference (WC) charts. Hypertension was defined by using the National Heart, Lung, and Blood Institute’s recommended cut points, and dyslipidemia was defined according to the recent recommendations of the American Heart Association.

**Results:**

The overall adjusted prevalences of “at risk for overweight” and overweight changed from 13% and 8% (using Iranian cutoffs), respectively, and 14.8% and 4.7% (using IOTF criteria) in 1999–2001 to 19% and 15% (Iranian cutoffs) and 23.0% and 9.2% (IOTF criteria) in 2006–2008 (*P* < 0.01 for all comparisons). The prevalence of abdominal obesity increased in males from 14.5% in 1999–2001 to 33.3% in 2006–2008 (*P* < 0.001). Almost half the adolescents had low high-density lipoprotein cholesterol (HDL-C) in the 3 surveys. In all surveys, as BMI and WC increased, multivariate age- and sex-adjusted odds ratios of low HDL-C and high triglyceride levels significantly increased. Overweight was associated with a greater likelihood of these risk factors, as compared with increased WC.

**Conclusions:**

Overweight and abdominal obesity are increasing in Tehranian adolescents, and these increases are accompanied by abnormalities in levels of serum triglyceride and HDL-C.

## INTRODUCTION

Atherosclerotic heart disease is one of the most important causes of morbidity and mortality,^1^ and its prevalence is escalating much more rapidly in developing countries.^2^ In contrast to trends in Northern Europe and the United States, 1999 data showed that mortality from cardiovascular disease (CVD) is increasing in Iran.^2^^,^^3^ Evidence from epidemiologic, pathologic, clinical, and genetic studies suggests that atherosclerosis begins during childhood.^4^^,^^5^ Obesity in childhood and adolescence often tracks into adulthood and results in increased incidence of subsequent metabolic syndrome, which is associated with cardiovascular disease and several cancers.^6^ Obesity and metabolic diseases in adolescence may also cause psychosocial and economic problems.^7^ The increasing prevalence of childhood obesity is a worldwide trend and has been observed among children and adolescents in the United States, Europe, Asia, and especially the Middle East.^2^^,^^8^^–^^10^ In Iran, a cross-sectional national survey of students aged 6 to 18 years documented prevalences of overweight and obesity of 8.8% and 4.5%, respectively.^11^

Previous reports revealed higher triglyceride (TG) and lower high-density lipoprotein cholesterol (HDL-C) levels in Iranian adolescents as compared with their counterparts in the United States and other countries.^2^^,^^12^^,^^13^ However, little is known regarding secular trends in serum lipid levels and obesity in adolescents. Therefore, the objectives of this study were to evaluate trends in overweight, abdominal obesity, hypertension, and dyslipidemia in Tehranian adolescents in 3 cross-sectional surveys (1999–2008) and assess the likelihood of these risk factors with respect to obesity status in each survey.

## METHODS

### Study population

This study was conducted within the framework of the Tehran Lipid and Glucose Study (TLGS), a prospective study of the prevalence of noncommunicable diseases and their risk factors among Tehran’s urban population. Data from the TLGS will be used to develop population-based measures and lifestyle modifications to decrease the prevalence of diabetes mellitus and dyslipidemia.^14^^,^^15^ The design of the present study encompasses 3 major components: survey 1 was a cross-sectional prevalence study conducted from 1999 to 2001, and surveys 2 (2002–2005) and 3 (2006–2008) were prospective follow-up surveys. Multistage cluster sampling was used to randomly select people aged 3 years or older from district 13 of Tehran, the capital of Iran. This population is served by 3 medical health centers. The age distribution of the population in district 13 is representative of the overall population of Tehran (Iran National Census, 1996).^15^ In the present study, 5207 adolescents aged 10 to 19 years (3010 from the 1999–2001 survey, 1107 from the 2002–2005 survey, and 1090 from the 2006–2008 survey) were selected from the 3 cross-sectional surveys. The study was approved by the research ethics committee of the Research Institute for Endocrine Sciences, Shahid Beheshti University of Medical Sciences, and informed written consent was obtained from the parents of each subject.

### Health examination

Weight was measured using digital scales (Seca, Hamburg, Germany) and was recorded to the nearest 100 grams while the subjects were minimally clothed and without shoes. Height was measured in standing position, without shoes, using a tape measure while the shoulders were in a normal position. Body mass index (BMI) was calculated as weight in kilograms divided by height in meters squared, and waist circumference (WC) was measured at the level of the umbilicus. Using a standard mercury sphygmomanometer, a qualified physician measured blood pressure (BP) twice while the subject was in a seated position during physical examination, after 1 initial measurement to determine peak inflation level. The mean of the 2 measurements was defined as the participant’s blood pressure. On the basis of the circumference of the participant’s arm, an appropriate cuff (pediatric or regular) was chosen (4 different sizes).^16^ A fasting blood sample was drawn between 7:00 AM and 9:00 AM from all study participants after a 12- to 14-hour overnight fast. Blood samples were taken in the sitting position according to a standard protocol and centrifuged within 45 minutes of collection.^12^ All blood lipid analyses were done at the TLGS research laboratory on the day of blood collection. Analysis of samples was performed using a Selectra 2 autoanalyzer (Vital Scientific, Spankeren, Netherlands). Total cholesterol (TC) and TG were assayed using enzymatic calorimetric tests with cholesterol esterase/cholesterol oxidase and glycerol phosphate oxidase, respectively. HDL-C was measured after precipitation of apoB-containing lipoproteins with phosphotungstic acid. Low-density lipoprotein cholesterol (LDL-C) was calculated from the serum TC, TG, and HDL-C concentrations expressed in mg/dL using the Friedewald formula.^17^ LDL-C was not calculated when the TG concentration was greater than 400 mg/dL. The performance of the assay was measured after every 20 tests using the lipid control serums Percinorm (cat. no. 1446070; Boehringer Mannheim, Mannheim, Germany) and Percipath (cat. no. 171778; Boehringer Mannheim) for normal and pathologic ranges of biochemical indexes, respectively. Lipid standard (cat. no. 759350, calibrated for automated systems; Boehringer Mannheim) was used to calibrate the Selectra 2 autoanalyzer for each day of laboratory analysis. All samples were analyzed when internal quality control met the acceptable criteria. Inter- and intra-assay coefficients of variation were 2% and 0.5%, respectively, for TC and 1.6% and 0.6% for TG.^12^

### Definitions

At risk for overweight (≥85th and <95th percentile), overweight (≥95th percentile), and abdominal obesity (≥90th percentile) were defined according to Iranian BMI and WC percentile reference data.^11^^,^^18^ Overweight and obesity also were defined using the age- and sex-specific BMI cutoffs recommended by the International Obesity Task Force (IOTF).^19^ To ensure uniformity of terms throughout the manuscript, the expressions “overweight” and “obesity” (recommended by IOTF criteria) were replaced by “at risk for overweight” and “overweight”. High systolic/diastolic BP was defined as values ≥95th percentile for sex, age, and height, ie, the recommended cut points of the National Heart, Lung, and Blood Institute. Dyslipidemia was defined according to the recent recommendations of the American Heart Association, ie, TC ≥200 mg/dL (5.2 mmol/L) and/or TG ≥200 mg/dL (2.26 mmol/L) and/or LDL-C ≥130 mg/dL (3.38 mmol/L) and/or HDL-C <40 mg/dL (1.04 mmol/L).^20^^,^^21^

### Statistical analysis

All statistical analyses were performed using SPSS (ver 16.0), and a *P* value less than 0.05 was considered significant. Comparisons of the means of normal variables among the 3 survey periods and between 2 groups were performed using ANOVA and the *t*-test in each age category (10–14 and 15–19 years). The Kruskal-Wallis test was used for non-normal variable (triglyceride). The χ^2^ test was used to examine differences in the prevalence of dyslipidemia, abdominal obesity, at risk for overweight, and overweight among data from the 3 surveys, and the Cochran-Armitage test was used to assess significance in the trends of these variables across the 3 survey periods.^22^ In the trend analyses, prevalence of variables was standardized to the 5-year age and sex distribution reported in the 2006–2007 Iranian census (Statistical Pocketbook of the Islamic Republic of Iran 2006–2007).^23^ Age- and sex-adjusted logistic regression was used separately in each survey to assess the likelihood of high lipid profiles and hypertension with respect to abdominal obesity and overweight.

## RESULTS

**Figure 1.**
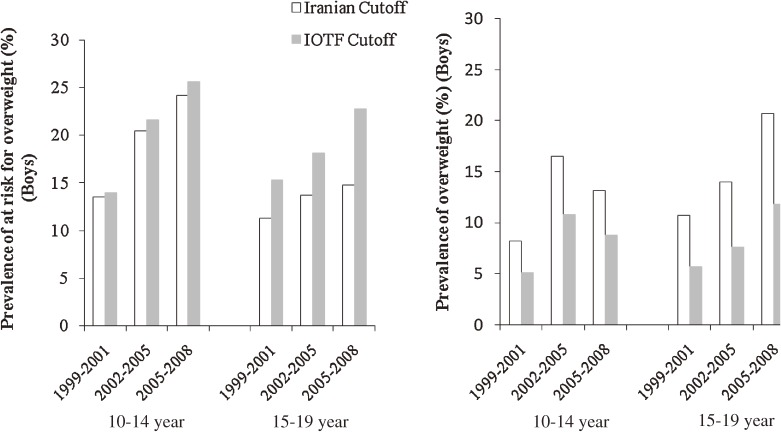
Adjusted prevalence and trend of at risk for overweight and overweight in adolescent males, according to Iranian BMI (body mass index) percentiles (≥85th and <95th percentile, ≥95th percentile; respectively) and IOTF (International Obesity Task Force) cutoffs in 2 age groups (age 10–14 and 15–19 years) during 3 time periods (1999–2008).

**Figure 2.**
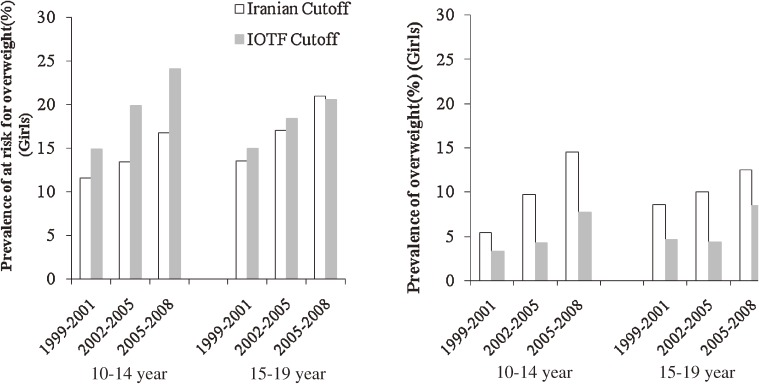
Adjusted prevalence and trend of at risk for overweight and overweight in adolescent females, according to Iranian BMI (body mass index) percentiles (≥85th and <95th percentile, ≥95th percentile; respectively) and IOTF (International Obesity Task Force) cutoffs in 2 age groups (age 10–14 and 15–19 years) during 3 time periods (1999–2008).

**Figure 3.**
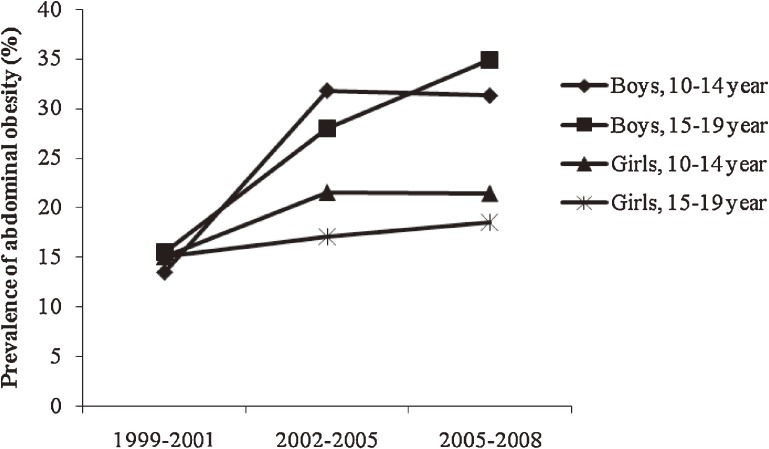
Adjusted prevalence and trend of abdominal obesity according to Iranian percentiles by age group (10–14 and 15–19 years) and sex during 3 time periods (1999–2008).

The three cross-sectional surveys comprised adolescents, among whom 1422 (47.2%), 536 (48.4%), and 508 (46.6%) were male in surveys 1, 2, and 3, respectively. In surveys 1, 2, and 3, nearly 63%, 67%, and 61% of subjects, respectively, had 1 or more risk factors. Among males aged 10 to 14 years and 15 to 19 years, mean (SD) BMI increased from 18.7 (4) and 21.5 (4) kg/m^2^ in 1999–2001 to 19.7 (5) and 23.0 (6) kg/m^2^ in 2006–2008, respectively (*P* < 0.01). Mean WC also increased both age groups (*P* < 0.01; Table 1). Among females aged 10 to 14 years and 15 to 19 years, mean (SD) BMI increased from 18.9 (4) and 21.7 (4) kg/m^2^ in 1999–2001 to 20.1 (4) and 22.6 (4) kg/m^2^ in 2006–2008, respectively (*P* < 0.01; Table 2). Overall, mean LDL-C was lower than 100 mg/dL during 2002–2008. Mean systolic/diastolic BP, HDL, LDL, and TC all decreased from 1999–2001 to 2006–2008 in both age groups and both sexes. The overall adjusted prevalence of at risk for overweight and overweight increased from 13% and 8% (Iranian cutoffs) and 14.8% and 4.7% (IOTF criteria), respectively, in 1999–2001 to 19% and 15% (Iranian cutoffs) and 23.0% and 9.2% (IOTF criteria) in 2006–2008 (*P* < 0.01; for all comparisons). Changes in the prevalence of at risk for overweight and overweight using Iranian and IOTF criteria from 1999–2001 to 2006–2008 are shown by age group and sex in Figures 1 and 2. The least change in the prevalence of overweight was seen in females aged 15 to 19 years. In males aged 15 to 19 years, the prevalence of abdominal obesity increased, and its prevalence in 2008 was nearly double that of 1999 (Figure 3). The prevalence of high diastolic BP decreased significantly among 10- to 14-year-old males (Table 3) and females (Table 4) over the 3 time periods. The prevalence of hypertriglyceridemia did not change during the 3 time periods in males (Table 3) or females (Table 4). The overall prevalence of low HDL-C was very high: nearly half of adolescents had low HDL-C. The prevalence of low HDL-C decreased significantly in females aged 10 to 14 years (Table 4). Overall, high LDL-C decreased by more than 50%, from 15.0% in 1999 to 7.4% in 2008 (*P* < 0.001). Mean LDL-C and the prevalence of hypercholesterolemia in survey 3 were significantly higher in females than in males in both age groups (*P* < 0.05). The Cochran-Armitage test revealed no significant trends in the prevalence of any investigated variable.

**Table 1.** Trends in anthropometric and metabolic characteristics in 2 age groups of adolescent males (1999–2008): Tehran Lipid and Glucose Study

	(1999–2001) Survey 1		(2002–2005) Survey 2		(2005–2008) Survey 3	

Age groups (years)	10–14	15–19	10–14	15–19	10–14	15–19
Age (years)^b^	12.4(1)	16.8(1)	12.2(1)	17.1(1)	12.2(1)	17.2(1)

*n* % adolescents in each survey	688 22.8	734 24.4	190 17.2	346 31.2	230 21.1	278 25.5

Weight (kg)	43.0(13)^b^	63.2(14)	47.2(15)	66.9(15)	43.3(15)^c^	68.1(16)^d^
Height (cm)	150.1(11)	171.1(7)	151.3(11)	172.4(6)	147.1(10)^c^	172.0(8)^d^
BMI^a^ (kg/m^2^)	18.7(4)	21.5(4)	20.2(5)	22.4(5)	19.7(5)^c^	23.0(6)^d^
WC^a^ (cm)	64.9(11)	73.9(11)	72.6(13)	80.4(12)	71.3(13)^c^	81.5(13)^d^
Systolic BP^a^ (mm Hg)	103(11)	110(12)	101(11)	108(11)	98(12)^c^	108(11)^d^
Diastolic BP^a^ (mm Hg)	69.5(9)	72.3(9)	65.6(10)	69.6(10)	63.8(10)^c^	69.0(10)^d^
TG^a^ (mg/dL)	89.0	97.0	96.5	92.0	82.0	91.0^d^
HDL-C^a^ (mg/dL)	45.0(11)	40.4(9)	42.3(11)	37.6(9)	46.8(11)^c^	39.8(8)^d^
LDL-C^a^ (mg/dL)	102(27)	98(30)	99(28)	88(25)	95(25)^c^	83(22)^d^
TC^a^ (mg/dL)	167(31)	161(35)	163(29)	145(29)	162(29)	144(27)^d^

^a^BMI: body mass index, WC: waist circumference, BP: blood pressure, TG: triglyceride, HDL-C: high-density lipoprotein cholesterol, LDL-C: low-density lipoprotein cholesterol, TC: total cholesterol. ^b^Means (SD), TG is median. ^c^Significant difference between the 3 surveys in 10–14 year age group. ^d^Significant difference between the 3 surveys in 15–19 year age group.

**Table 2.** Trends in anthropometric and metabolic characteristics in two age groups of adolescent females (1999–2008): Tehran Lipid and Glucose Study

	1999–2001 Survey 1		2002–2005 Survey 2		2005–2008 Survey 3	

Age groups (years)	10–14	15–19	10–14	15–19	10–14	15–19
Age (years)^b^	12.3(1)	17.0(1)	12.3(1)	17.3(1)	12.3(1)	17.2(1)

*n* % adolescents in each survey	675 22.4	913 30.4	214 19.3	357 32.3	231 21.2	351 32.2

Weight (kg)	43.6(11)^b^	55.5(10)	45.2(12)	56.9(11)	44.4(12)	57.6(11)^d^
Height (cm)	150.9(9)	159.5(5)	150.6(8)	159.7(6)	147.5(9)^c^	159.4(5)
BMI^a^ (kg/m^2^)	18.9(4)	21.7(4)	19.7(4)	22.2(4)	20.1(4)^c^	22.6(4)^d^
WC^a^ (cm)	66.9(9)	72.5(9)	68.5(10)	73.3(9)	66.9(10)	71.3(9)^d^
Systolic BP^a^ (mm Hg)	102(11)	106(11)	97.0(11)	101(10)	96.0(12)^c^	99.0(11)^d^
Diastolic BP^a^ (mm Hg)	70.0(10)	72.9(9)	66.0(10)	69.2(8)	62.3(10)^c^	65.6(9)^d^
TG^a^ (mg/dL)	107.0	91.5	101.0	81.0	95.0^c^	84.0^d^
HDL-C^a^ (mg/dL)	42.5(10)	43.9(10)	41.1(10)	41.1(10)	43.7(10)^c^	44.2(10)^d^
LDL-C^a^ (mg/dL)	104(28)	104(27)	95(24)	95(26)	95(25)^c^	91(26)^d^
T-Chol^a^ (mg/dL)	171(31)	169(31)	159(29)	153(28)	160(30)^c^	154(31)^d^

^a^BMI: body mass index, WC: waist circumference, BP: blood pressure, TG: triglyceride, HDL-C: high-density lipoprotein cholesterol, LDL-C: low-density lipoprotein cholesterol, TC: total cholesterol. ^b^Mean (SD), TG is median. ^c^Significant difference between the 3 surveys in 10–14 year age group. ^d^Significant difference between the 3 surveys in 15–19 year age group.

**Table 3.** Prevalence and trend of metabolic abnormalities in adolescent males (1999–2008): Tehran Lipid and Glucose Study

	(1999–2001) Survey 1		(2002–2005) Survey 2		(2005–2008) Survey 3	

Age groups (years)	10–14	15–19	10–14	15–19	10–14	15–19
Age (year) Mean (SD)	12.4(1)	16.8(1)	12.2(1)	17.1(1)	12.2(1)	17.2(1)

*n* %	688 22.8	734 24.4	190 17.2	346 31.2	230 21.1	278 25.5

High systolic BP^a^ (%)	4.8	2.5	1.1	0.9	2.7	0.4^c^
High diastolic BP^a^ (%)	11.3	4.6	7.4	1.8	5.4^d^	3.3
High TG^b^ (%)	5.3	9.3	7.9	5.5	4.0	8.9
Low HDL-C^b^ (%)	36.1	55.5	44.9	64.3	25.2^d^	54.1
High LDL-C^b^ (%)	15.1	11.7	12.1	5.7	8.8^d^	3.6^c^
High TC^b^ (%)	14.2	12.4	10.7	4.0	8.5^d^	3.7^c^

^a^High systolic/diastolic blood pressure (BP) was defined as ≥95th percentile for sex, age, and height from the National Heart, Lung, and Blood Institute’s recommended cut points. ^b^Dyslipidemia was defined according to the recent recommendations of the American Heart Association, ie, total cholesterol (TC) ≥200 mg/dL (5.2 mmol/L) and/or triglyceride (TG) ≥200 mg/dL (2.26 mmol/L) and/or low-density lipoprotein cholesterol (LDL-C) ≥130 mg/dL (3.38 mmol/L) and/or high-density lipoprotein cholesterol (HDL-C) <40 mg/dL (1.04 mmol/L). ^c^Significant difference between the 3 surveys in 15–19 year age group. ^d^Significant difference between the 3 surveys in 10–14 year age group.

**Table 4.** Prevalence and trend of metabolic abnormalities in adolescent females (1999–2008): Tehran Lipid and Glucose Study

	1999–2001 Survey 1		2002–2005 Survey 2		2005–2008 Survey 3	

Age groups (years)	10–14	15–19	10–14	15–19	10–14	15–19
Age (years) Mean (SD)	12.3(1)^b^	17.0(1)	12.3(1)	17.3(1)	12.3(1)	17.2(1)

*n* %	675 22.4	913 30.4	214 19.3	357 32.3	231 21.2	351 32.2

High systolic BP^a^ (%)	3.9	1.3	1.6	0.6	2.3	0.6
High diastolic BP^a^ (%)	12.3	4.8	7.0	2.1	3.2^c^	0.9^d^
High TG^b^ (%)	9.4	4.7	8.8	1.7	5.7	4.1
Low HDL-C^b^ (%)	44.3	40.2	48.8	47.4	35.5	35.9
High LDL-C^b^ (%)	14.9	17.6	9.1	11.0	10.1^c^	7.7^d^
High TC^b^ (%)	15.9	16.0	9.8	6.6	11.8	8.7^d^

^a^High systolic/diastolic blood pressure (BP) was defined as ≥95th percentile for sex, age, and height from the National Heart, Lung, and Blood Institute’s recommended cut points. ^b^Dyslipidemia was defined according to the recent recommendations of the American Heart Association, ie, total cholesterol (TC) ≥200 mg/dL (5.2 mmol/L) and/or triglyceride (TG) ≥200 mg/dL (2.26 mmol/L) and/or low-density lipoprotein cholesterol (LDL-C) ≥130 mg/dL (3.38 mmol/L) and/or high-density lipoprotein cholesterol (HDL-C) <40 mg/dL (1.04 mmol/L). ^c^Significant difference between the 3 surveys in 15–19 year age group. ^d^Significant difference between the 3 surveys in 10–14 year age group.

**Figure 4.**
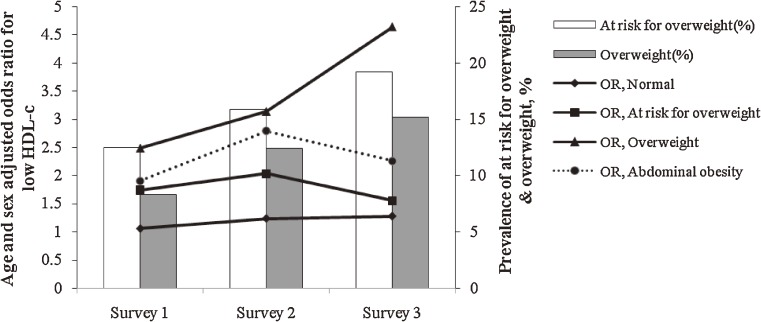
Prevalence of at risk for overweight and overweight; age- and sex-adjusted odds ratios (ORs) for low HDL-C in adolescents aged 10 to 19 years, Tehran Lipid and Glucose Study: 1999–2001 (Survey 1), 2002–2005 (Survey 2), and 2006–2008 (Survey 3). An increase in body mass index (BMI) from “normal” to “at risk for overweight” and “overweight” and increase in waist circumference (WC) from normal to “abdominally obese” significantly increased the likelihood of low HDL-C in the 3 cross-sectional surveys. BMI increments had a greater impact on the likelihood of these risk factors in survey 3 than in the other 2 surveys. BMI had a greater impact on these risk factors than did WC.

**Figure 5.**
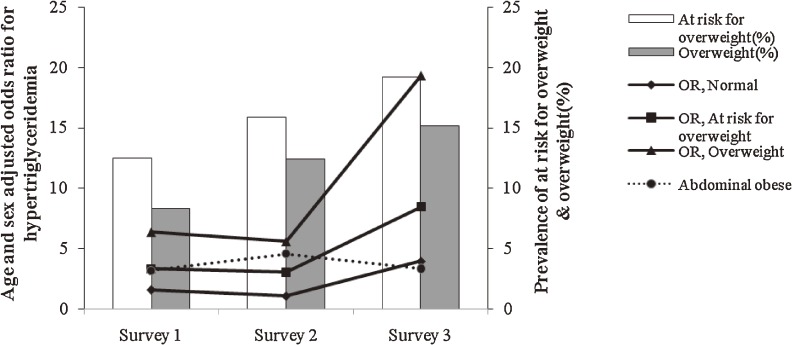
Prevalence of at risk for overweight and overweight; age- and sex-adjusted odds ratios (ORs) for hypertriglyceridemia in adolescents aged 10 to 19 years, Tehran Lipid and Glucose Study: 1999–2001 (Survey 1), 2002–2005 (Survey 2), and 2006–2008 (Survey 3). An increase in body mass index (BMI) from “normal” to “at risk for overweight” and “overweight” and increase in waist circumference (WC) from “normal” to “abdominally obese” significantly increased the likelihood of hypertriglyceridemia in the 3 cross-sectional surveys. BMI increments had a greater impact on the likelihood of these risk factors in survey 3 than in the other 2 surveys.

Both BMI and WC significantly increased the likelihood of low HDL-C (Figure 4) and high TG levels (Figure 5) in the 3 cross-sectional surveys, and, as compared with abdominal obesity and at risk for overweight, overweight was associated with higher odds ratios for these risk factors. In survey 3, an incremental increase in BMI was associated with higher odds ratios for these risk factors as compared with the 2 other surveys.

As BMI increased from normal to at risk for overweight and overweight, the multivariate age- and sex-adjusted odds ratios were 1, 1.24, 2.46, and 3.90 (*P* < 0.01) for high LDL-C and 1, 1.32, 3.42, and 5.37 (*P* < 0.01) for TC in 1999–2001; these likelihoods were not significant in 2002–2005 or 2006–2008. As WC increased from normal to abdominally obese, the odds ratios for high systolic and diastolic BP were 2.12 and 2.65, respectively, in survey 1 (*P* < 0.01), 3.17 and 13.04 in survey 2 (*P* < 0.01), and 2.07 (*P* < 0.01) and 1.58 (nonsignificant) in survey 3.

## DISCUSSION

The present study analyzed data from 3 cross-sectional surveys and revealed that, according to Iranian percentiles and IOTF criteria, the prevalence of overweight increased in Tehranian adolescents in 2 age groups in a recent 9-year period. The prevalences of overweight and abdominal obesity were lower among females than among males. Females aged 15 to 19 years showed less increase in the prevalence of at risk for overweight, overweight, and abdominal obesity than did their peers aged 10 to 14 years. The prevalence of abdominal obesity doubled in males, and the risk of hypertension, high LDL-C, and TC decreased, as compared with their peers in 1999–2001. However, the prevalence of high TG was unaltered, and almost half the adolescents had low HDL-C in the 3 time periods. Overweight increased the likelihood of high TG and low HDL-C to a greater extent than did high WC, and the odds of having these risk factors increased from 1999–2001 to 2006–2008. Nearly two thirds of all adolescents had at least 1 metabolic abnormality in each survey.

To our knowledge, this is the first study to report trends in adolescent obesity and dyslipidemia in Iran. Childhood obesity has evolved into a worldwide epidemic,^24^^,^^25^ and as 26% to 63% of obese children will become obese adults,^26^ this epidemic will not subside unless aggressive public heath campaigns against obesity are implemented.

The use of international criteria facilitates comparison among studies of overweight prevalence and trends. However, international criteria (IOTF) underestimate the prevalence of adolescent overweight among Asians due to ethnic differences, which could delay implementation of prevention programs.^27^ Further research is needed to explore BMI patterns in children from Africa and Asia.^19^

The prevalence of overweight in NHANES (1999–2004)^28^ was higher than in the present study, but the rate of increase was higher in the present population, especially in males. It has been reported that the prevalence of overweight is much higher in the Middle East as compared with other developing countries.^2^^,^^29^ Although childhood obesity might be related to specific cultural and regional circumstances, it is also affected by social factors, exercise, advertising, public policies, and rapid urbanization.^2^

Considering the increasing trend in overweight seen in our study, we anticipated a similar increase in hypertension; however, this was not seen, in conformity with the downward trend noted in other studies.^8^^,^^30^^,^^31^ The downward pattern in high SBP and DBP in our study is similar to that described among young adults in the United Kingdom and Australia.^32^ In the Minneapolis Children’s BP Study of 10- to 14-year-old children,^33^ SBP percentiles were significantly higher and DBP percentiles were significantly lower in 1996 than in 1986, whereas in the Bogalusa study,^34^ both SBP and DBP were lower at the end of the study periods (1975 to 1981 and 1984 to 1992), despite an increase in obesity in both cohorts. However, NHANES (1963 to 2002) indicated that, among US children and adolescents, elevated BP has been increasing since the late 1980s.^8^ Moreover, it has been shown that after a long decreasing trend, BP begins to rise approximately 10 years after an increase in obesity. In addition, elevated BP is correlated with small body size at birth, which results from reduced growth throughout gestation^35^; hence, current improvements in the nutrition and health of girls and young women may be a cause of this decreasing trend in BP.

Contrary to our expectations, the adolescents in the present experienced a decrease in TC and LDL-C cholesterol. In NHANES I (1971–1974) and NHANES III (1988–1994), adolescents experienced the same decreasing trend in TC^36^; however, in some other countries increasing trends in blood lipids were seen.^37^^,^^38^ The lack of a rapid increase in blood lipids despite the rising prevalence of overweight suggests that other factors such as nutrition and reduced physical activity may have an influence.^39^^,^^40^ There is much variation in specific cholesterol levels by age, sex, and race among different populations and even the same population over a period of time; however, a pattern of an early increase followed by a decrease in mean values of cholesterols from preadolescence to adolescence is very common,^41^^,^^42^ and is similar to the trend observed in our study.

As compared with American adolescents,^39^ serum TG was higher and HDL-C was lower despite the lower prevalence of overweight in the present subjects. Previous studies of Tehranian adolescents showed that approximately 6.5% of all adolescents and nearly 39% of overweight adolescents met the criteria for hypertriglyceridemic waist phenotype.^43^ Also, in Korean adolescents, the most common feature of the metabolic syndrome was hyperglycemia followed by low HDL-C and hypertriglycemia.^44^ In addition, rising overweight is strongly related to concentrations of HDL-C and TG, but is only weakly associated with TC and LDL-C concentrations. Park et al found that the prevalence of metabolic syndrome remains high among obese Korean, Japanese, and Chinese adolescents.^44^ The impact of abdominal obesity on the prevalence of these risk factors is minimal, which suggests that the decreasing trend in LDL-C, TC, and BP may be due to latent factors.

The National Food Consumption Survey (1995) showed lower intakes of whole grains among Iranians (<2 servings per day). Esmaillzadeh et al observed that intake of refined grains was associated with higher odds of hyperglycemia and hypertension^45^ and that bread grains were associated with the lowest dietary diversity score in that population, probably due to culture, dietary habits, and the limited number of bread-grain products (eg, whole grain cereals, fortified macaroni, and whole grain biscuits) as compared with developed countries. Dietary diversity scores of bread-grain and dairy groups in females were higher than in males.^46^ It has been shown that breakfast and daily exercise are negative risk factors for atherogenic changes in childhood,^38^ and any effect of weight increase on risk factors for cardiovascular disease should have manifested itself during the 8- to 9-year interval.^39^ Hence, for better control of weight and other risk factors among adolescents, research to identify behavioral factors, such as diet composition and physical activity, is needed.

The present study is limited by the fact that several factors, including muscle-to-fat ratio, timing of the adolescent growth spurt, and sexual maturation, are potentially confounding variables that were not measured. However, the median ages of sexual organ growth and pubic hair growth in males were 9.01 and 10.34 years, respectively, and the median ages of breast growth and pubic hair growth among females were 9.74 years and 10.49 years, respectively, which accord with other studies of Tehranian adolescents.^47^ The median age of menarche in females was 12.6 years.^47^^,^^48^ Onset of puberty seems to be earlier among Iranian males than among those from other countries, but onset of secondary pubertal characteristics in females is similar to that in other white females; therefore, a comparison with other studies is feasible in females. In this study, change in serum lipid profile due to puberty was a reason for age classification and better control of this confounding factor. Future studies with more extensive data may be able to provide additional information on trends in risk factors of CVD.

### Conclusion

Overweight and abdominal obesity are increasing among Tehranian adolescents, and these increases seriously affect their TG and HDL-C levels. The reasons for the lack of deleterious effects on other CVD risk factors are not known. Nevertheless, it is clear that these obese youths are likely to become obese adults with higher risks of chronic diseases occurring at earlier ages, in combination with increased morbidity and mortality. Therefore, monitoring of trends in obesity and the potential effects of CVD risk factors is vital in designing programs that target prevention of CVD, which must begin with the development of healthy lifestyles in childhood.
